# Comparative Mitogenomic Analysis of Species Representing Six Subfamilies in the Family Tenebrionidae

**DOI:** 10.3390/ijms17060841

**Published:** 2016-05-31

**Authors:** Hong-Li Zhang, Bing-Bing Liu, Xiao-Yang Wang, Zhi-Ping Han, Dong-Xu Zhang, Cai-Na Su

**Affiliations:** 1 School of Life Sciences, Datong University, Datong 037009, China; lyl_kgy@126.com (H.-L.Z.); 15935231706@163.com (Z.-P.H.); intron.mayfly@163.com (D.-X.Z.); 2 Institute of Loess Plateau, Shanxi University, Taiyuan, Shanxi 030006, China; liubb2013@gmail.com; 3 College of Life Science, Shaanxi Normal University, Xi’an 710062, China; 18032396815@163.com

**Keywords:** Tenebrionidae, mitogenome, RNA, AT-rich region, phylogeny

## Abstract

To better understand the architecture and evolution of the mitochondrial genome (mitogenome), mitogenomes of ten specimens representing six subfamilies in Tenebrionidae were selected, and comparative analysis of these mitogenomes was carried out in this study. Ten mitogenomes in this family share a similar gene composition, gene order, nucleotide composition, and codon usage. In addition, our results show that nucleotide bias was strongly influenced by the preference of codon usage for A/T rich codons which significantly correlated with the G + C content of protein coding genes (PCGs). Evolutionary rate analyses reveal that all PCGs have been subjected to a purifying selection, whereas 13 PCGs displayed different evolution rates, among which ATPase subunit 8 (*ATP8*) showed the highest evolutionary rate. We inferred the secondary structure for all RNA genes of *Tenebrio molitor* (*Te2*) and used this as the basis for comparison with the same genes from other Tenebrionidae mitogenomes. Some conserved helices (stems) and loops of RNA structures were found in different domains of ribosomal RNAs (rRNAs) and the cloverleaf structure of transfer RNAs (tRNAs). With regard to the AT-rich region, we analyzed tandem repeat sequences located in this region and identified some essential elements including T stretches, the consensus motif at the flanking regions of T stretch, and the secondary structure formed by the motif at the 3′ end of T stretch in major strand, which are highly conserved in these species. Furthermore, phylogenetic analyses using mitogenomic data strongly support the relationships among six subfamilies: ((Tenebrionidae incertae sedis + (Diaperinae + Tenebrioninae)) + (Pimeliinae + Lagriinae)), which is consistent with phylogenetic results based on morphological traits.

## 1. Introduction

The mitogenome is becoming an important information resource for many research fields, such as phylogenetics, population genetics, phylogeography, and comparative and evolutionary genomics due to its simple genomic organization, the lack of recombination, and high rates of evolution in comparison to nuclear genome [1,2,3,4]. The insect mitogenome is a small double-stranded circular molecule with lengths ranging from 14,000 to 20,000 bp, usually encoding 13 protein coding genes (PCGs), including 3 cytochrome *c* oxidase subunits (*COX 1*-*3*), cytochrome b (*CYTB*), 2 ATPase subunits (*ATP6* and *ATP8*), 7 nicotinamide adenine dehydrogenase (NADH) subunits (*ND1*-*6* and *ND4L*), 2 ribosomal RNAs (*16S rRNA* and *12S rRNA*), and 22 transfer RNAs (tRNAs) [5,6]. In addition to 37 coding genes, the mitogenome contains a large non-coding region that is rich in adenine (A) and thymine (T) (also known as the AT-rich region in insects). This region usually contains essential regulatory elements for initiating and regulating the replication and transcription of the mitogenome [5,6]. With the development of the next generation of sequencing technology, complete mitogenome sequences have been increasing dramatically in recent years. Large amounts of mitogenomes have been extensively used for addressing deep-level phylogenetic relationships [7,8,9]. In addition to application in phylogeny, the mitogenome can provide a number of genome-level characters.

Tenebrionidae, a large family in Coleoptera, contains more than 25,000 species from 12 subfamilies and is widely distributed on a worldwide basis [10]. They may be from a wide variety of different areas, including gulf, desert, cropland, forest, and grassland environments. Their feeding habit is very complex and most of them are important agricultural pests. In comparison with the rich Tenebrionidae species, information about the Tenebrionidae mitogenome is still limited. To date, only 10 complete mitogenomes have been sequenced for Tenebrionidae (GenBank, 8 April 2016), of which two mitogenomes are from two specimens of *Tenebrio molitor*, three mitogenomes are from three specimens of *Tribolium castaneum*, and the remaining mitogenomes are from one representative specimen of five species, respectively (Table 1). Despite all that, it is very important to gather lots of minimum but significative genetic information from sequenced mitogenomes of present species, considering that many living organisms are facing the threat of extinction due to some natural factors. Furthermore, the mitogenome owns a great deal of interesting evolutionary features such as nucleotide composition, codon usage, nucleotide substitution, and transfer RNAs (tRNAs) and ribosomal RNAs (rRNAs) secondary structures [11,12,13]. These features can only be obtained from comparative analyses. In addition, because the annotation of part of the mitochondrial genes is error prone, we can adjust delimitation of genes by comparison with that from closely related species.

In this paper, we compared ten mitogenomes representing six subfamilies of Tenebrionidae in detail, including nucleotide composition, PCGs, conserved regions in tRNAs and rRNAs, and structural elements in AT-rich regions. We refined the original annotation of some genes. These revises and detailed comparative analyses will not only enrich our understanding of the comparative biology of mitogenomes from Tenebrionidae species, but also provide unique insights into the evolution of Tenebrionidae at the genomic level. Finally, we reconstructed the phylogeny of the six subfamilies based on different partitioned datasets from mitogenomes.

## 2. Results

### 2.1. Nucleotide Composition

The nucleotide composition is AT biased in all ten Tenebrionidae mitogenomes, ranging from 69.7% in *Asbolus verrucosus* to 73.6% in *T.* *audax*. By comparative analyses of the nucleotide compositions of four major partitions (PCGs, rRNAs, tRNAs, AT-rich region), we found all four partitions are consistently biased towards A and T, among which, PCGs always displayed the lowest A + T content, whereas the highest A + T content was found in the AT-rich region in ten Tenebrionidae specimens. In addition, the A + T content of three codon sites in PCGs were also analyzed; we found the third codon sites had a higher A + T content than the first and second codon sites (Supplementary Table S1).

Furthermore, we measured the strand bias in the nucleotide composition by AT- and GC-skews. Ten Tenebrionidae mitogenomes displayed consistent AT- and GC-skews, the PCGs in the major strand displayed slight A- or T-skews and moderate C-skews, whereas the PCGs in the minor strand displayed marked T-skews and G-skews. These strand biases are analogous with the patterns found in most metazoan mitogenomes [14]. A marked T-skew was present in the second codon site of PCGs on both the coding strand and the third codon site of PCGs in the minor strand, whereas the third codon site of PCGs in the major strand displayed marked A-skews. The AT-rich region, the second and the third codon sites of PCGs in the major strand displayed marked C-skews, whereas a significantly biased G-skew was found in rRNAs, tRNAs in the minor strand, and the first and the third codon sites of PCGs in the minor strand (Supplementary Tables S2 and S3).

In addition, we analyzed the nucleotide composition in 13 PCGs and found A + T content is low in four genes (*COX1*, *COX2 COX3*, and *CYTB*), whereas the highest A + T content was found in *ATP8*, *ND4L*, and *ND6*. All PCGs in the major strand are C-skewed and the three genes (*ATP8*, *COX2*, *ND2*) are slightly A-skewed, whereas all four PCGs encoded in the minor strand are T-skewed and G-skewed (Supplementary Tables S4–S6).

### 2.2. Protein-Coding Genes (PCGs)

#### 2.2.1. Initiation and Termination Codons

In a few PCGs of the ten Tenebrionidae mitogenomes, the position of the initiation or termination codons were redefined. Most PCGs in these Tenebrionidae mitogenomes initiated with a typical ATN codon, in addition, TTG was also proposed as the initiation codon for *ND3* in *Adelium* sp. and *ND5* in *U*. *dermestoides* because it has been suggested that TTG, GTG, and GTT can also be used as the initiation codon for PCGs in invertebrate mitogenomes (Supplementary Table S7) [15]. Several kinds of codons (AAC, ATA, AAT, and TCT) were proposed as the initiation codon for *COX1*. The initiation codon of *COX1* in insect mitogenomes has been frequently discussed, because it is difficult to identify the typical ATN start codon at the beginning of the open reading frame after trnY, which is mainly attributed to the lack of research on an explicit RNA expression of *COX1* for most insect orders.

Most PCGs terminated with the canonical TAN (N represents any one of four nucleotides, A, T, C, G) stop codon in ten Tenebrionidae mitogenomes, in which TAA was more frequently used than TAG. The incomplete stop codon T/TA was often found in three COX genes and two ND genes (*ND4* and *ND5*) in most of the ten Tenebrionidae specimens (Supplementary Table S8); this type of codons may be the product of the selective pressure to economize the mitogenome size [16]. In addition, we found tRNA was arranged at the downstream of all these PCGs with truncated termination codons. The partial termination codon was frequently found in insect mitogenomes, which has been presumed to be corrected by post-transcriptional polyadenylation [17].

#### 2.2.2. Codon Usage

The four codons, UUU (Phe), UUA (Leu), AUU (Ile), and AUA (Met) were the most frequently used codons in all ten sequenced Tenebrionidae mitogenomes with values ranging from 21.7% in *A.* *verrucosus* to 29.2% in *T. audax* (Supplementary Table S9), which play a key role for the high A + T content in whole mitogenome. Furthermore, the usage proportion of the four AT-rich codons seem to be positively correlated to the A + T content of PCGs (*R*^2^ = 0.87) in ten Tenebrionidae specimens (Supplementary Figure S1), as reported in other insects [18].

**Figure 1:**
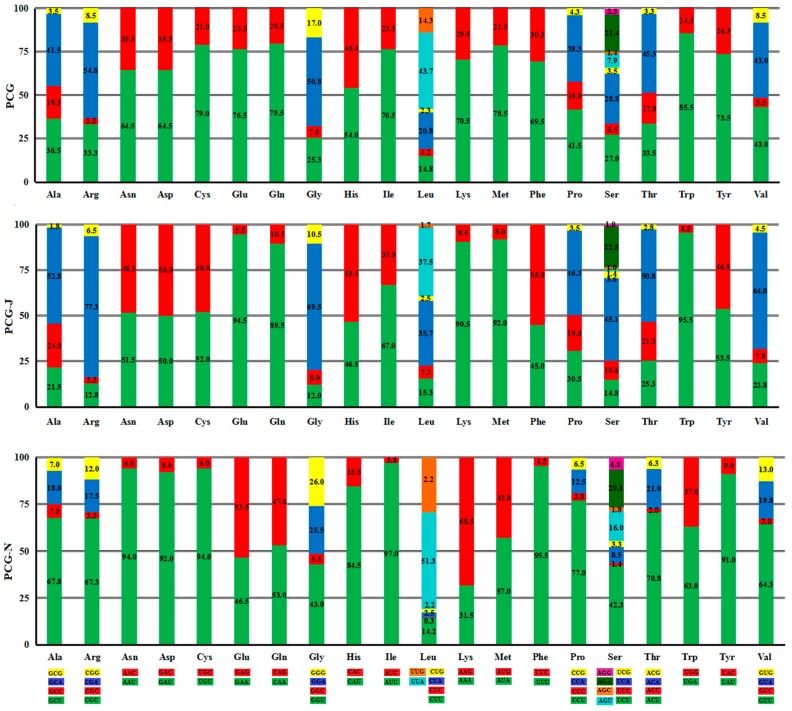
Percentage of synonymous codon usage of each amino acid in the ten Tenebrionidae mitogenomes. Codon families were provided on the *x*-axis.

In all PCGs from the ten Tenebrionidae mitogenomes, NNG (N represents any one of four nucleotides, A, T, C, G) (8.8%) was the least used codon, whereas NNA (N represents any one of four nucleotides, A, T, C, G) (39%) was the most used codon; PCGs in the major strand also presented the same bias. Nevertheless, in PCGs encoded by the minor strand, NNC (N represents any one of four nucleotides, A, T, C, G) (3.2%) was the most infrequently used codon and NNU (N represents any one of four nucleotides, A, T, C, G) (54.5%) was the most frequently used codon (Supplementary Table S10). The codons that ended with adenine (A) were strongly biased to be utilized in the fourfold degenerate codons of PCGs in the major strand. Whereas, the fourfold degenerate codon usage presented a bias towards uridine (U) in the third codon site of PCGs in the minor strand, except the Ser (AGN), in which the codons that ended with A were used most frequently. With regard to the twofold degenerate codons, the codons that ended with A/U were biased to be used on both strands, except for Lys and Glu in the minor strand, which show the preference for G rather than A (Figure 1).

**Figure 2:**
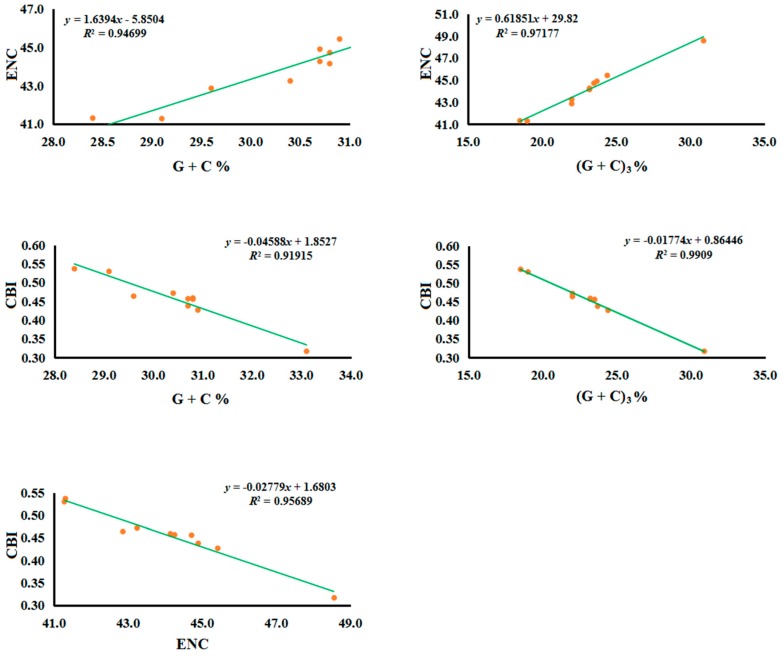
Evaluation of codon bias across ten Tenebrionidae mitogenomes. ENC denotes effective number of codons; CBI denotes codon bias index; G + C denotes GC content of codons; (G + C)_3_ denotes GC content of the third codon positions.

To further evaluate the codon usage bias in the ten Tenebrionidae mitogenomes, we analyzed the correlations between the codon bias index (CBI), the effective number of codons (ENC), the G + C content of all codons (G + C), and the G + C content of the third codon sites ((G + C)_3_) (Figure 2). We found ENC seems to be strongly positive correlated with the G + C content for either all codons (*R*^2^ = 0.95) or the third codon sites (*R*^2^ = 0.97) (Figure 2). In contrast, CBI displayed a strongly negative correlation with ENC (*R*^2^ = 0.96), G + C content for either all codons (*R*^2^ = 0.92), and the third codon sites (*R*^2^ = 0.99) (Figure 2).

#### 2.2.3. Gene Evolutionary Rate

**Figure 3:**
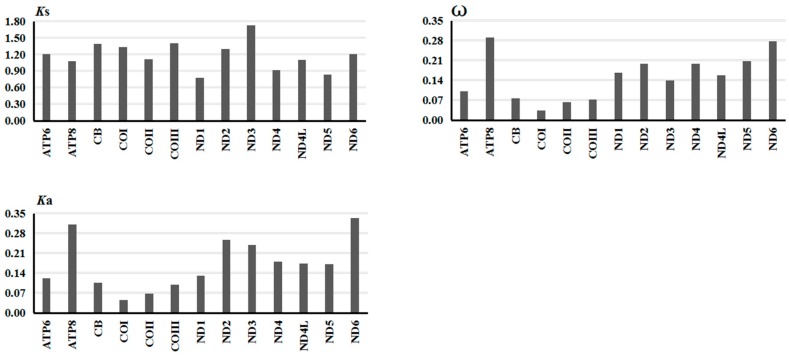
Evolutionary rates of 13 protein-coding genes in the ten Tenebrionidae mitogenomes. *K*s denotes Synonymous nucleotide substitutions per synonymous site; *K*a denotes nonsynonymous nucleotide substitutions per nonsynonymous site; ω denote the ratio of *K*a/*K*s.

To analyze the evolutionary patterns of PCGs in the ten Tenebrionidae mitogenomes, the rates of non-synonymous substitutions (*K*a), the rates of synonymous substitutions (*K*s), and the *K*a/*K*s ratio (ω) were calculated for each PCG, respectively. In all PCGs, *ND3* showed the highest *K*s, *ND6* showed the highest *K*a, whereas *ATP8* showed the highest ω values. Four genes (*COX1*-*3* and *CYTB*) uniformly displayed the low ω, indicating that there are strong evolution constraints in these genes (Figure 3). Notably, the *K*a/*K*s ratios for all mitochondrial PCGs were below 0.3, which may mean that all PCGs were evolving under a strong purifying selection. Therefore, we could combine all mitochondrial PCGs to analyze the phylogeny of the six subfamilies in Tenebrionidae. Furthermore, we found the ω value is negatively correlated with the G + C content of the mitochondrial PCGs (*R*^2^ = 0.86) (Supplementary Figure S2), which indicate that G + C content may be one important element in determining the evolutionary patterns of mitochondrial PCGs.

### 2.3. Transfer RNAs (tRNAs)

All 22 canonical tRNAs in invertebrate mitogenomes were found in all ten Tenebrionidae mitogenomes. The average length ranges from 62 ± 1.3 to 71 ± 0.5 bp (Supplementary Table S11). In the *T. molitor* mitogenome, all tRNAs could be folded into the typical cloverleaf secondary structure with the exception of trnS^AGN^, in which its dihydrouridine (DHU) arm was replaced by a DHU-loop. The percent of identical nucleotides (%INUC) was calculated for each tRNA family of the ten Tenebrionidae mitogenomes. Notably, the strand bias was observed in the conservation pattern of tRNA genes. Six tRNAs showed the highest conservative level (%INUC > 75%); only one of them was encoded by the minor strand. Four tRNAs (*trnY*, *trnD*, *trnF*, and *trnH*) showed more varying sites, in which the identical percent of nucleotides is just close to 50% and only *trnD* was encoded by the major strand (Supplementary Table S11). Thus, the conservation of tRNA genes in the ten Tenebrionidae mitogenomes is markedly biased to the major strand.

**Figure 4:**
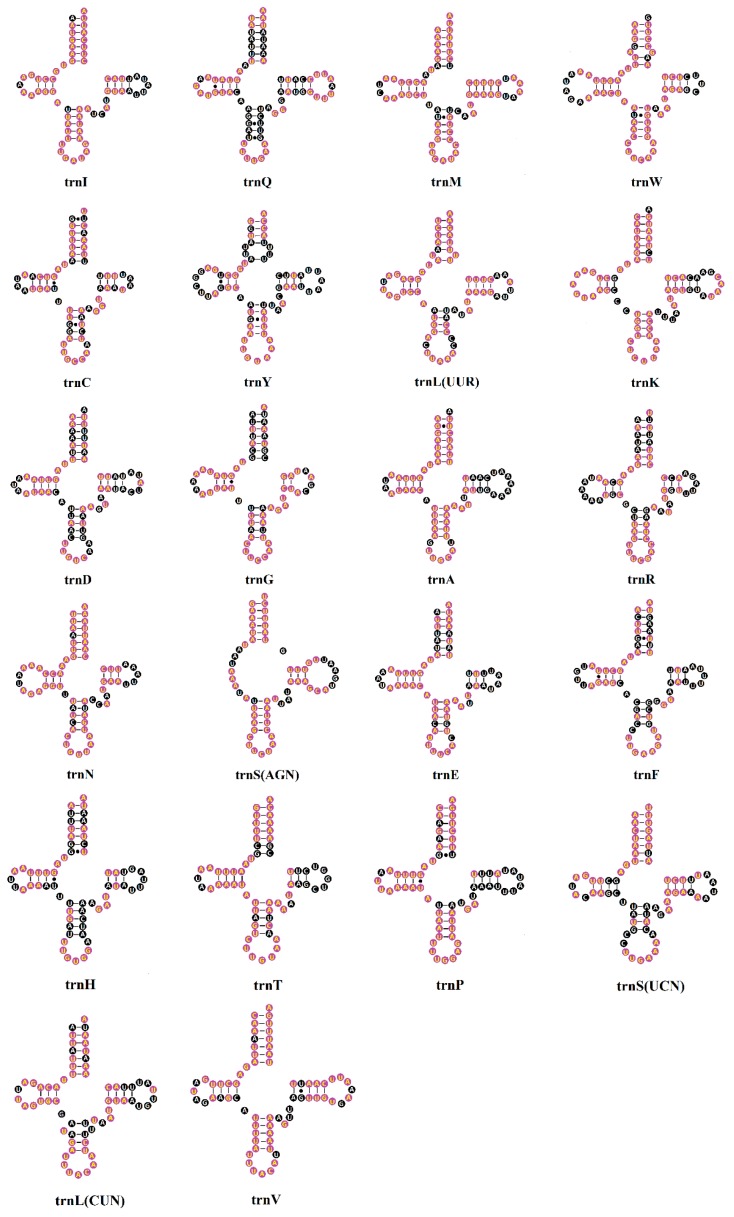
Inferred secondary structures of 22 transfer RNAs (tRNAs) identified in the *T. molitor* mitogenome. Completely conserved sites within the ten Tenebrionidae specimens were marked by yellow nucleotides within purple spheres. Bars denote Watson-Crick base pairings, and dots denote G-U base pairings.

Furthermore, completely conserved sites of each tRNA within ten Tenebrionidae mitogenomes were present in the secondary structure. The stem part plays an important role in maintaining the stability of the tRNAs’ secondary structure. By comparative analyses, we found these nucleotides in the stem of the tRNAs are actually more conserved (>60%) than those in loops, except for the AC loop. In four arms of the tRNAs’ secondary structure, nucleotides in the aminoacyl acceptor stem (AA stem) and DHU arm are relatively more conserved (>70%) than those in the pseudouridine arm (TψC arm) and anticodon arm (AC arm) (Figure 4; Supplementary Table S12). Among the four loops, only the anticodon loop were extremely conserved (92.86%), and most of the variable nucleotides were mainly present in the TψC loop (21.63%). In addition, we found all tRNAs have 2 bp located at the junction between the AA stem and AC arm except for *trnL^CUN^* which has 1 bp at the same site (Figure 4). Surprisingly, the nucleotides in this site in most tRNAs are highly conserved among the ten Tenebrionidae specimens.

### 2.4. Ribosomal RNAs (rRNAs)

**Figure 5:**
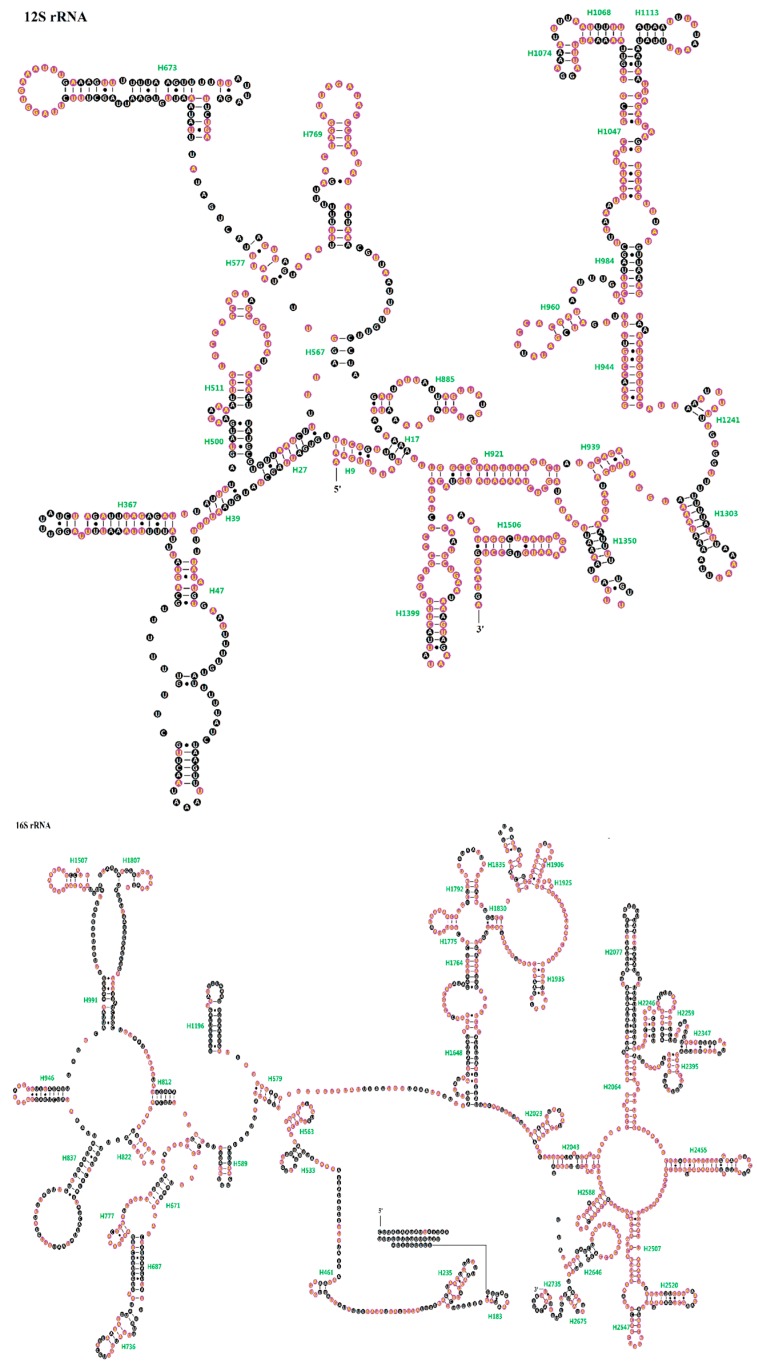
Inferred secondary structure for two ribosomal RNAs (*12S rRNA* and *16S rRNA*) in the *T. molitor* (Te2) mitogenome. Completely conserved sites within the ten Tenebrionidae specimens were marked by yellow nucleotides within a purple sphere. Bars denote Watson-Crick base pairings, and dots denote G-U base pairings.

The *16S rRNA* was located between *trnL^CUN^* and *trnV*, and the *12S rRNA* was located at the downstream of *trnV*. In this study, we redefined the boundaries of *12S rRNA* in all ten Tenebrionidae mitogenomes. The length of *16S rRNA* ranges from 1277 bp in *T.* *confusum* to 1288 bp in *T. audax*, whereas *12S rRNA* has a length ranging from 747 bp in *A.* *verrucosus* to 764 bp in *U.* *dermestoides* (Supplementary Table S11). The secondary structure of *16S rRNA* from the *T. molitor* mitogenome was derived by following the mitochondrial *rrnL* model for *Drosophila* *melanogaster*, while the secondary structure of *12S rRNA* was inferred according to the model for *D. virilis* [19,20]. The secondary structures of two *T. molitor* (*Te2*) rRNAs were displayed as the representative, in which completely conservative sites in ten Tenebrionidae mitogenomes were marked. The secondary structure of *12S rRNA* could also be subdivided into four structural domains (I–IV) and 27 helices (Figure 5), whereas *16S rRNA* consisted of five canonical structural domains (I–II, IV–VI) and 44 helices, because the domain III degenerated to a length of single strand as the link between domain II and domain IV (Figure 5).

In *12S rRNA*, domains III and IV are more conservative than domains I and II either in sequence or the secondary structure among the ten Tenebrionidae specimens. There are eight helices in domain I (H9–H511), the 5′ end of this domain consists of a pseudoknot formed by H9 and H17, in which the stem H9 is highly conserved (90.00%), though the nucleotide G was substituted by A in *T. confusum* and *T. audax* which actually more stabilized the last couplet of H9. Besides H9, H511 in this domain is also relatively conserved (80.77%) (Supplementary Table S13). H47 is highly variable among most insects and it is difficult to propose one relatively conserved structure for this region [21]. The possible structure of this region presumed for *T. molitor* consisted of two long stems, one short stem, and three loops, and the three stems were divided by the front large loop, not similar to some insects in which this helix consists of a long stem [22,23]. Domain II consisted of five helices (H567–H885) and is highly variable among the ten Tenebrionidae specimens, especially in the helix H673. Nevertheless, the loop abutting H673, the distal part of H769, as well as the loop abutting H769 are extremely conserved (100%). There are 12 helices (H921–H1350) in domain III, the helices H921–H960 and the most part of H1047 are relatively conserved among the ten Tenebrionidae specimens (>75%) (Supplementary Table S13). In domain III, the most instable portion is the distal part of the helix H1047, which may be attributed to its high AT content. In most insects, the helix H1068 consists of a six base-pair-long stem [24]. The six couplets in H1068 were observed in *T. molitor*, which also could be formed in other Tenebrionidae species and the two couplets at both ends are extremely conserved. Thus, the helix comprises 5′-AA XX AU-3′ on one side and 5′-AU XX UU-3′ on the other side, two nucleotides in the middle on both sides can also be matched in all ten Tenebrionidae specimens. Although two nucleotides on one side of H1074 are not highly conserved, three canonical base pairs could be found in this helix for the ten Tenebrionidae specimens. Domain IV only contains two helices, H1399 and H1506, which are relatively conserved both in sequence (>75%) and structure (Figure 5; Supplementary Table S13).

In *16S rRNA*, domains IV and V are more conserved in the ten Tenebrionidae specimens than the other three domains. Domain I contained only five helices and was aligned ambiguously, nonetheless, H563 is highly conserved in the ten Tenebrionidae specimens (100%). There are 14 helices (H579–H1807) in domain II, among which these helices H736, H777, and H1507 are relatively conserved (>75%) (Supplementary Table S14). The loop abutting several helixes (H777, H822, H946, H1507, and H1807) are also highly conserved. Domain IV contains nine helices (H1648–H1935), all helices are highly conserved in ten Tenebrionidae specimens except for three helixes (H1648, H1764, and H1935) (Supplementary Table S14). Five couplets were observed in H1792, similar as found in most insects [25], and the first couplet in this helix often formed a non-canonical U-U interaction. There are 13 helices (H2023–H2588) in domain V, in which the secondary structure of most helices are highly conserved except for H2077, H2246, H2259, and H2347. The helix H2077 is the most complicated region and difficult to align, in fact, consistent structure could not be found for this region. Domain VI contains three helices, in which only H2735 and the loop abutting H2646 are extremely conserved (100%) (Figure 5; Supplementary Table S14).

### 2.5. Non-Coding Regions

37 genes in the ten Tenebrionidae mitogenomes were arranged highly economized. Except for the AT-rich region, the long intergenic gap between *trnS^UCN^* and *ND1* was found in all ten Tenebrionidae specimens and the size and nucleotide sequence are relatively conserved in this region (Supplementary Table S15). Except for *U.* *dermestoides*, the ten Tenebrionidae specimens have 17 bp in this gap and the sequence “ATACTAAATTTTATTAA” is conserved among most of the ten Tenebrionidae mitogenomes—only a single nucleotide mutation was found in a few species (*Adelium* sp., *T. confusum* and *Tribolium audax*) (Supplementary Figure S3). This gap in *U. dermestoides* is 18 bp in size and the nucleotide sequence is slightly variable compared with others. Even so, when this region was aligned across the ten Tenebrionidae mitogenomes, we found one common motif (TACTAA) (Supplementary Figure S3).

**Figure 6:**
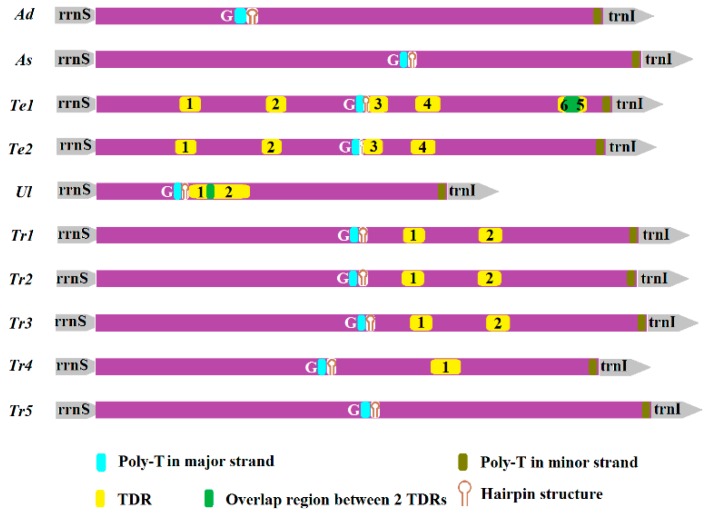
Organization of the AT-rich region in ten Tenebrionidae mitogenomes. Tandem repeats (TDRs) refers to tandem repeats.

The AT-rich region was located between *12S rRNA* and *trnI* in all ten Tenebrionidae mitogenomes, as found in most insects. The size is varying in this region of ten Tenebrionidae mitogenomes, ranging from 798 in *U.* *dermestoides* to 1266 bp in *T. audax* (Supplementary Table S15). This region in mitogenome is also called the control region, which can be classified into two groups in insects: group 1 contained one highly conserved domain and one variable domain, while group 2 contained a few short conserved sequence blocks [26]. According to detailed analyses, we found the control region in the ten Tenebrionidae specimens belong to neither the first nor the second group in insects. By comparison, some essential elements could be identified for the ten Tenebrionidae control region (Figure 6).

The presence of tandem repeats (TDRs) in the mitochondrial AT-rich region has been frequently reported in insects. In the ten Tenebrionidae mitogenomes, we only found some short tandem repeats in this region of four species (*T. molitor*, *U. dermestoides*, *T. castaneum*, *T. confusum*), ranging from 14 to 44 in size. By comparison of 11 repetitive sequences, we found TDRs occur in a random order in the AT-rich region of the ten Tenebrionidae specimens; nevertheless, they may be slightly inclined to happen at the middle and end of this region. Most TDRs were found to repeat one time and displayed different sequence homology between repeat units, ranging from 74% in Tr_1 to 90% in Te_1. In addition, all analyzed TDRs are consistently biased towards being AT rich. The AT content of the repeat motifs in most TDRs were greater than 90% except for Te_1 (AT% = 85%) (Table 2). Two *T. molitor* specimens harbored four common AT-rich repetitive regions, while in *Te1*, two other repetitive regions (Te1_5 and Te1_6) were found (Figure 6). Te1_5 overlapped 34 nucleotides with Te1_6. The difference in this region between two individuals may be explained by the lack of correlation between the rate of point mutations and the rate of insertion/deletion among this section [26]. In addition, overlap between TDRs also existed in the two repetitive regions (Ul_1 and Ul_2) in *U. dermestoides*.

The ten Tenebrionidae specimens exhibited perfect T stretch longer than ten nucleotides in the middle of the AT-rich region, which were found in the major strand (Table 3). *Adelium* sp. even exhibits two stretches (22 and 10 bp, respectively) at this location. By comparing the flanking regions of the T stretch, we found that the T stretch (22 bp) is more similar to that found in other Tenebrionidae species. In the minor strand, the T stretch (over 10 bp) was found only in a few species. When the minimum size of the T stretch was diminished to 8bp, the T stretch located in the minor strand can be identified from the remaining species (Table 3). However, there are three problems in these T stretches in the minor strand. Firstly, two or more T stretches were found in some species (e.g., three in *A. verrucosus* and two in *U. dermestoides*). Secondly, the position of the T stretch is random, either at the beginning, middle, or end of the AT-rich region in the ten Tenebrionidae specimens. Thirdly, we could not find the conserved flanking regions surrounding these T stretches. According to these reasons, T stretches we found are not homologous with that near the trnIle gene, which is considered as one of the conserved elements of insect control regions and related to mtDNA replication origin [26]. By further aligning the sequence near trnIle in the minor strand, we found that this section at the primarily beginning of the AT-rich region is relatively conserved, and the sequence is rich in thymine (Supplementary Figure S4). In *T. molitor* and *T. confusum*, seven continued thymine nucleotides were found, while in other Tenebrionidae species, a single thymine in the middle or end of this T stretch was always substituted by other nucleotides.

**Figure 7:**
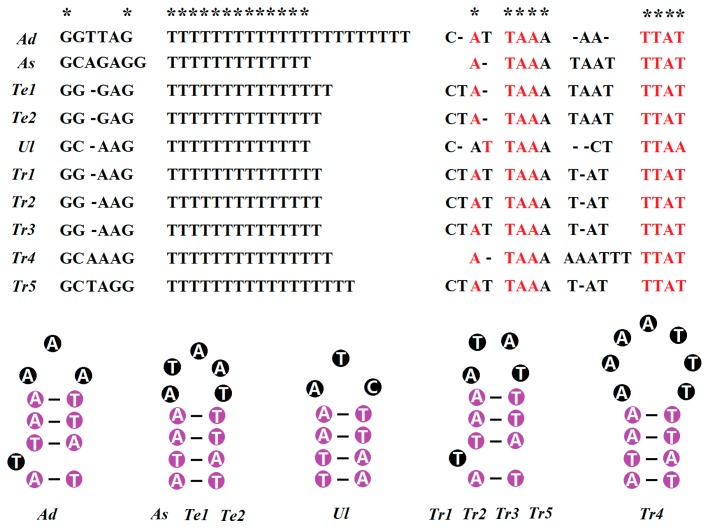
T stretch in the major strand, the abutting flanking regions, and the stem and loop structure formed by the 3′ flanking region in ten Tenebrionidae mitogenomes. * denotes the conserved site. Red letters denote the nucleotide located in the stem of stem—loop structure, white letters with purple circle as background denote nucleotides in the four conserved couplets of the stem, white letters with black circle as background denote variable nucleotides in the stem—loop structure.

The flanking regions of two T stretches are relatively conserved. The consensus motif “GTAA” was found at the 3′ end of the T stretch (in the minor strand) in most of the ten Tenebrionidae specimens. The “AAAXC” motif is present at the 5′ end of this stretch in the ten Tenebrionidae specimens (Figure 7). The conserved motif was not found at the 5′ end of the T stretch (in the major strand); the flanking region is just rich in G. At the 3′ end of this T stretch, the conserved motif “A(T)TAAAX_n_TTA(A)T” was found (Figure 7). Furthermore, microsatellite “(AT)_(n≥5)_” was detected in all ten Tenebrionidae specimens, and the largest microsatellite only harbors eight consecutive AT. These microsatellites were mainly located in the middle portion of AT-rich regions (Table 3).

Overall, ten Tenebrionidae AT-rich regions show some distinct/consensus sequences and structural characteristics, such as differentiated tandem repetitions, T stretches, flanking regions, stem and loop structures. These features may be used as important genetic markers for studies of Tenebrionidae.

### 2.6. Phylogenetic Implications

**Figure 8:**
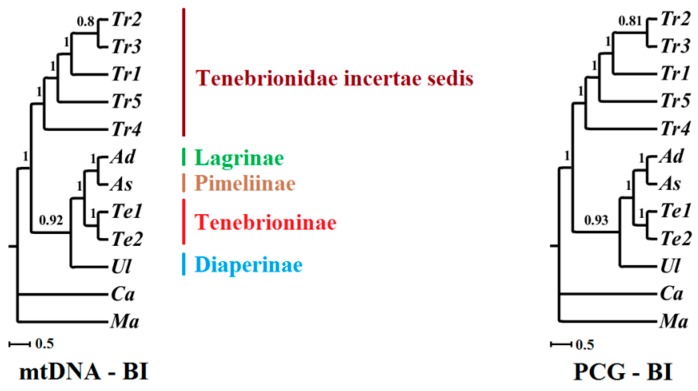

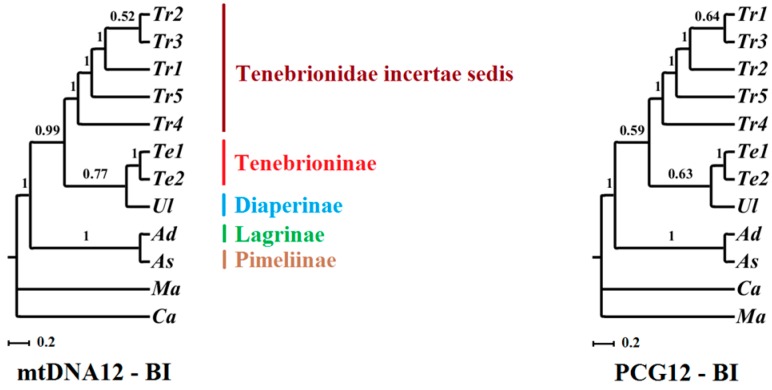
Phylogeny of the six subfamilies in Tenebrionidae. Phylogenetic trees were inferred from four datasets by using Bayesian inference (BI) method. Applicable bootstrap values were shown close to the nodes.

Our analyses based on four datasets consistently recover the sister group relationship between Pimeliinae and Lagriinae, the monophyly of Tenebrionidae incertae sedis, and the genetic relationship within this genus with high support. In Tenebrionidae incertae sedis, thee *T. castaneum* is firstly grouped with *T. audax*, then grouped together with *T. confusum*, which is consistent with the phylogenetic results based on morphology and other molecular markers [27,28]. Nonetheless, the tree topologies based on four datasets displayed different phylogenetic relationships among the six subfamilies. In our analyses based on mtDNA and PCG, Tenebrionidae incertae sedis formed an independent clade, and the other four subfamilies Pimeliinae, Lagriinae, Diaperinae, and Tenebrioninae grouped together and formed the other independent clade, and occupied the basal position of Tenebrionidae. Within the second clade, our analyses strongly support a relationship of Diaperinae + Tenebrioninae + (Pimeliinae + Lagriinae), which seems to be different from the traditional taxonomy. Nonetheless, our analyses based on mtDNA12 and PCG12 support the sister group relationship between Diaperinae and Tenebrioninae, which subsequently is grouped together with Tenebrionidae incertae sedis, whereas the minor subdivision made up of Pimeliinae and Lagriinae was placed as the basic position among six subfamilies (Figure 8), which is also supported by the morphological taxonomy [27,28].

## 3. Discussion

### 3.1. Mitochondrial Coding Genes

According to statistical analysis of the relative synonymous codon frequencies (RSCU) for PCGs from ten Tenebrionidae specimens, we found that the codons that ended with A/T were biased to be used than those that ended with G/C in either four- or two-fold degenerate codon usage. Furthermore the usage pattern of codons in PCGs displayed nucleotide bias with the preference for those codons rich in A and T. By further analyses, we found the codon usage biases were mainly determined by the G + C content as mentioned in the neutral mutational theories [29].

In 13 mitochondrial PCGs, A + T content was the highest in *ATP8*, which also displayed the highest evolutionary rate, and three *COX* and *CYTB* genes owning low A + T content displayed slow evolutionary rate, which may suggest that the evolutionary rate of PCG is positively correlated with its A + T content.

By comparatively analyzing the conserved and variable sites of tRNAs and rRNAs in ten Tenebrionidae specimens, we found some important conserved stem, loop, and junctions in the cloverleaf structure of tRNAs. Furthermore, conserved helixes and loops were found in each domain of the secondary structure of rRNAs. These conserved regions may play key roles in the formation of the secondary structure, three-dimensional structure, and functions for RNAs. The structural information may help us to refine the alignment of rRNA sequences more accurately in phylogenetic analyses [30].

### 3.2. Non-Coding Regions

By comparative analyses on ten sequenced Tenebrionidae mitogenomes, some important common features were identified. The common motif (TACTAA) was found in the intergenic gap between *trnS^UCN^* and *ND1*, which is similar with the corresponding conserved motif (ATACTAA) in Lepidoptera [21]. Furthermore, the consensus sequence has been suggested as the possible binding site of the transcription termination factor, since the intergenic gap located near the end site of the coding region in the major strand is in the circular mitogenome [31].

With regard to the AT-rich region of the ten Tenebrionidae specimens, we found some tandem repeats in four species. The origin of tandem repeats may be explained by different models, but the slippage-strand mispairing during mtDNA replication seems to be the primary mechanism causing the occurrence of tandem repeat units in the mitogenome [32,33]. We found that some repeat unit sequences could be folded into stem-loop secondary structures (Supplementary Figure S5), which were thought to be facilitating the capability to generate tandem repeats [33]. On the other hand, these secondary structures may promote replication slippage by stabilizing the slipped strand or blocking the polymerase [34]. Furthermore, we found the repeat units of most TDRs displayed different sequence homology. The homogenization or divergence between repeats mainly depends on the relative rate of copy addition and deletion (copy turnover) to the nucleotide mutation rate. If the rate of copy turnover is less relative to the rate of nucleotide substitution, then the copies in the array will diverge [32].

We found some TDRs with overlap regions in two Tenebrionidae species. The repeat region including Te1_5 and Te1_6 in *Te1* may be explained by three ways: (1) The repeat unit of Te1_5 (RU5) tandemly repeated 1.9 times; (2) The repeat unit of Tel_6 (RU6) repeated one time and then RU5 located in RU6 repeated one time again; (3) RU6 repeated two times, while when this motif was repeated in the second time, seven nucleotides in the 5′ end of RU6 were lost, which may be more logical in explaining this repeat region. The overlapping range between Ul_1 and Ul_2 is relatively small and just harbors 12 nucleotides (Figure 6), which may be explained by the way that the repeat unit of Ul_1 (RU1) is repeated 2.6 times and then RU2 (including part of RU1) is repeated one time.

T stretches longer than 10 nucleotides in the AT-rich region were considered to be essential for the initiation of mtDNA replication in insects [35]. By search and comparison, we found common T stretches (more than 10) in the middle of this region in the major strand in all ten Tenebrionidae specimens (Table 3). A short sequence rich in thymine near trnIle was also found in the minor strand in these Tenebrionidae specimens (Supplementary Figure S4). It has been suggested the relatively conserved T stretch on both coding strands may be involved in the replication initiation of mtDNA for the opposite strand [36]. The consensus and conserved motif were found at two ends of a T stretch (in the minor strand). One conserved motif was also found at the 3′ end of a T stretch (in the major strand), which can form one hairpin structure for ten Tenebrionidae specimens. Although there are some variations of nucleotides, the structural integrity of this structure is generally maintained. The stem consisted of four perfect matching nucleotides, while the number and the type of nucleotides in the loop is variable, from three in *U. dermestoides* to seven in *T.* *confusum* (Figure 7). This stem and loop structure might be important for the replication of circular DNA molecules, since the replication origin for the minor strand was considered to be located upstream of the T stretch in the middle portion of the CR [37]. Alternatively, they could play a role in regulatory functions during the transcription of mtDNA.

### 3.3. Proper Dataset in Phylogenetic Analyses

Phylogenetic analyses among six subfamilies in Tenebrionidae were performed using four datasets by the Bayesian inference method. A dataset seems to be one significant factor in determining the phylogenetic tree topology. It has been suggested that phylogenetic reconstructions in some taxa may be strongly affected by the third codon sites. In this study, we used four datasets and found the resolution in our analyses was improved when the third codon sites were completely removed (mtDNA12 and PCG12). In addition, some advantages including commonness and richness in all organelles, single copy in each genome, no spacer region, and the conservation in sequence and structure have made rRNAs good models for studying the phylogenetic relationships at different taxonomic levels [38]. It has been proposed that 12S rRNA and 16S rRNA in the mitogenome are more suitable for analyzing the phylogeny between genus, species group, and those species earlier in divergence time. Actually, tRNA genes have also been verified as a better molecular marker for resolving the phylogeny at a relatively low level [39]. While in this research, the tree topology have not been influenced by RNA genes, and the trees based on mtDNA or mtDNA12 are identical with those using PCG or PCG12, respectively. Nevertheless, our analyses with RNAs did change the branch support in trees (Figure 8).

Mitogenome has often been used for studying phylogenetic relationships at deeper taxonomic levels. Nonetheless, our results using mtDNA12 and PCG12 are considerably congruent with the traditional phylogenetic relationships analyzed based on morphological and molecular data, suggesting that mitogenome may also be used for resolving phylogenetic relationships among subfamilies within a family.

## 4. Methods

### 4.1. Bioinformatics Analysis

A few encoding genes were reannotated by alignment with the same gene from closely related species. All nucleotide compositions and codon usages were analyzed using the Mega 5.0 [40]. The strand asymmetry was analyzed by AT-skew and GC-skew; AT-skew = (A − T)/(A + T) and GC-skew = (G − C)/(G + C) were used as the formulas to measure these values [41]. The selective pressure that these PCGs in the mitogenome were evolving under were estimated using the ratio (Ka/Ks). Ks and Ka were determined using DnaSP [42]. In addition, this software package was also used to calculate ENC and CBI for all PCGs. We identified the tandem repeats in the AT-rich region by the tandem repeat finder [43] and inferred the possible hairpin structures of some TDRs or the region abutting T-stretches in the AT-rich region using the program Mfold [44].

### 4.2. Construction of Secondary Structures of RNAs

The secondary structure of 20 tRNAs in the *T. molitor* (*Te2*) mitogenome were predicted by using tRNAscan-SE 1.21 [45], and the remaining two (*trnR* and *trnS^AGN^*) were determined through alignment with those from closely related species. The secondary structure of *12S rRNA* and *16S rRNA* were inferred following the models predicted for *D. virilis* and *D. melanogaster*, respectively. Helix numbering was named following the convention established at the CRW site [46]. Those sections lacking homology were folded using Mfold [44].

### 4.3. Phylogenetic Analyses

The phylogenetic trees among six subfamilies in the family Tenebrionidae were reconstructed using mitogenome sequences of ten specimens from seven Tenebrionidae species. Two species, *Calosoma* sp. and *Macrogyrus oblongus* from Adephaga, were selected as outgroups (Table 1). Four datasets were constructed as follows: (1) PCGs (all protein coding genes); (2) mtDNA (all 37 coding genes); (3) PCG12; (4) mtDNA12, and the latter two are the same as the first two datasets, except for the removal of the third codon sites. All PCGs were individually aligned using MEGA 5.0 based on amino acid sequence alignments [40]. RNA genes were individually aligned with Clustal X 1.83 [47]. All individual gene alignments were then concatenated according to the need of datasets by using the software Bioedit 7.0 [48]. MrModeltest 2.3 was used to select the model for datasets [49]. The phylogenetic reconstruction using the Bayesian method was based on partitioned datasets by codons (first, second, and third) and RNA genes, so we presumed the model for four partitions, respectively. According to the Akaike information criterion, the GTR + I + G model was also selected as the optimal BI model for RNA genes, and the first and second codon sites, whereas the GTR was selected for the third codon sites. For the Bayesian analyses, we used MrBayes 3.1.2 with four MCMC chains running for one million generations [50]. Each set was sampled every 100 generations. The Bayesian consensus trees were generated after a burnin of 25% of the retained trees.

## 5. Conclusions

In the present study, we made comprehensive and systematical comparative analyses on ten mitogenomes representing six subfamilies in Tenebrionidae. Ten mitogenomes share identical gene composition and gene order. The nucleotide composition is AT biased in all ten mitogenomes, which was strongly influenced by the preference of codon usage for A/T rich codons. Evolutionary rate in 13 PCGs revealed that *ATP8* evolve at the fastest rate, which correlated with its higher A + T content. The conserved and variable sites in the secondary structure of tRNAs and rRNAs were analysed in ten Tenebrionidae specimens, some important conserved helices (stems) and loops were found. These conserved regions may play key role in the structure and functions of RNAs. With regard to the AT-rich region, T stretch was found in the major and the minor coding strand, respectively, and the consensus motifs were also found at the flanking regions of T stretches in ten Tenebrionidae specimens. Furthermore, phylogenetic analyses among six subfamilies suggest that mitogenome is one better data for resolving phylogenetic relationships among subfamilies within a family.

## Figures and Tables

**Table 1:** List of taxa used in this research.

Subfamily	Species	Accession Number	Abbreviation
Lagrinae	*Adelium* sp.	NC_013554	*Ad*
Pimeliinae	*Asbolus verrucosus*	NC_027256	*As*
Tenebrioninae	*Tenebrio molitor*	NC_024633	*Te1*
Tenebrioninae	*Tenebrio molitor*	KP994554	*Te2*
Diaperinae	*Ulomoides dermestoides*	NC_025332	*Ul*
Tenebrionidae incertae sedis	*Tribolium castaneum*	NC_003081	*Tr1*
Tenebrionidae incertae sedis	*Tribolium castaneum*	KM009121	*Tr2*
Tenebrionidae incertae sedis	*Tribolium castaneum*	KM244661	*Tr3*
Tenebrionidae incertae sedis	*Tribolium confusum*	NC_026702	*Tr4*
Tenebrionidae incertae sedis	*Tribolium audax*	NC_024600	*Tr5*
Outgroup (Carabinae)	*Calosoma* sp.	GU176340	*Ca*
Outgroup (Gyrinidae)	*Macrogyrus oblongus*	FJ859901	*Ma*

**Table 2:** Statistics of tandem repeat sequence in the AT-rich region from ten Tenebrionidae mitogenomes.

Species	TDRs	Consensus Size (bp)	Copy Number	Position in AT-Rich Region		AT%	Percent Matches	Stem-Loop	Average ΔG
*Te1*	Te1_1	20	2.2	192–236	5′	85.00	84	–	–
	Te1_2	22	1.9	389–430	M	100.00	85	–	–
	Te1_3	22	1.9	622–662	M	100.00	90	1	1.09
	Te1_4	24	2.3	730–783	M	95.83	83	1	−2.05
	Te1_5	16	2.9	1070–1118	3′	93.75	77	–	–
	Te1_6	24	2	1057–1103	3′	87.50	84	–	–
*Te2*	Te2_1	20	2.2	183–227	5′	85.00	84	–	–
	Te2_2	22	1.9	380–421	M	100.00	85	–	–
	Te2_3	22	1.9	612–652	M	100.00	90	1	1.09
	Te2_4	24	2.3	720–773	M	95.83	83	1	−2.05
*Ul*	Ul_1	14	3.6	215–267	5′	100.00	75	1	−0.50
	Ul_2	44	2.1	255–350	M	97.73	86	2	−4.40
*Tr1*	Tr1_1	17	2.8	702–748	M	100.00	81	–	–
	Tr1_2	18	2.7	875–924	3′	100.00	74	–	–
*Tr2*	Tr2_1	17	2.8	700–746	M	100.00	81	–	–
	Tr2_2	18	2.7	873–922	3′	100.00	74	–	–
*Tr3*	Tr3_1	17	2.8	719–765	M	100.00	81	–	–
	Tr3_2	18	2.7	892–941	3′	100.00	74	–	–
*Tr4*	Tr4_1	20	3.2	767–831	3′	95.00	75	–	–

5′, M, and 3′ denote the 5′ end, the middle part, and the 3′ end of AT-rich region in major strand, respectively. TDRs: tandem repeats; ΔG: Gibbs free energy.

**Table 3:** T stretch in both coding strands and the microsatellite AT in major strand of the AT-rich region from ten Tenebrionidae mitogenomes.

Species	T Stretch					Microsatellite AT		
	Position in Major Strand	Position in Minor Strand				Positions (Size) in Major Strand		
*Ad*	319–340	290–299				381–392 (6)	433–444 (6)	
*As*	696–708	138–147	887–901	1211–1222	1233–1242	801–816 (8)	782–793 (6)	
*Te1*	595–609	173–192				633–646 (7)	749–760 (6)	860–869 (5)
*Te2*	586–599	173–192				623–636 (7)	739–750 (6)	850–859 (5)
*Ul*	180–192	21–30	104–113			250–265 (8)	464–475 (6)	285–294 (5)
*Tr1*	582–595	794–802				718–727 (5)		
*Tr2*	580–593	793–800				716–725 (5)		
*Tr3*	599–612	793–800				735–744 (5)		
*Tr4*	508–522	51–58	868–875			770–783 (7)	812–821 (5)	791–800 (5)
*Tr5*	606–622	69–76				746–755 (5)		

## Supplementary Materials

Supplementary materials can be found at http://www.mdpi.com/1422-0067/17/6/841/s1.
