# Supplementary material for: Comparative Mitogenomic Analysis of Species Representing Six Subfamilies in the Family Tenebrionidae
Source: Int J Mol Sci. 2016 May 31;17(6):841. doi: 10.3390/ijms17060841 (PMC4926375; doi:10.3390/ijms17060841)
Supplement: Supplementary file 1 [file ijms-17-00841-s001.pdf]

Supplementary Materials: Comparative Mitogenomic Analysis of Species Representing Six Subfamilies in the Family Tenebrionidae

Hong-Li Zhang, Bing-Bing Liu, Xiao-Yang Wang, Zhi-Ping Han, Dong-Xu Zhang and Cai-Na Su

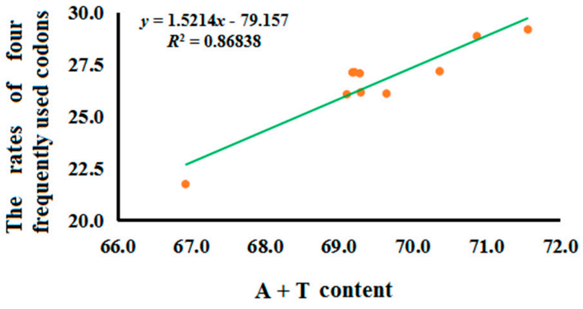

Figure S1. Correlation between the percentage of the four most frequently used codons and A + T content of all protein-coding genes.

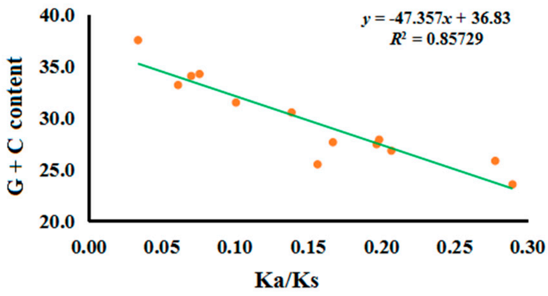

Figure S2. Correlation between ω and G + C content of protein-coding genes.

```
      ******    **  ****
Ad   -ATACTAATTTTTATTAA
As   -ATACTAAATTTTATTAA
Te1  -ATACTAAATTTTATTAA
Te2  -ATACTAAATTTTATTAA
Ul   AGTACTAAAAATTATTAA
Tr1  -ATACTAAATTTTATTAA
Tr2  -ATACTAAATTTTATTAA
Tr3  -ATACTAAATTTTATTAA
Tr4  -TTACTAAATTTTATTAA
Tr5  -GTACTAAATTTTATTAA
```

Figure S3. The sequence of the gap between *trnS^UCN^* and *ND1* in ten Tenebrionidae mitogenomes. Note: Nucleotides with red color denote the site of nucleotide mutation; * denote the completely conserved site.

```
      *** * ***  *   ****
Ad    AAA-C TTTTCTA  GTAA
As    AAATC TTTTTTG  ATAA
Te1   AAATC TTTTTTT  GTAA
Te2   AAATC TTTTTTT  GTAA
Ul    AAA-C TTTTCTT  ATAC
Tr1   AAAAC TTTATTT  GTAA
Tr2   AAAAC TTTATTT  GTAA
Tr3   AAAAC TTTATTT  GTAA
Tr4   AAAAC TTTTTTT  GTAA
Tr5   AAAAC TTTTTTA  GTAA
```

Figure S4. T stretch in the minor strand and the abutting flanking regions in ten Tenebrionidae mitogenomes. * denote the completely conserved site.

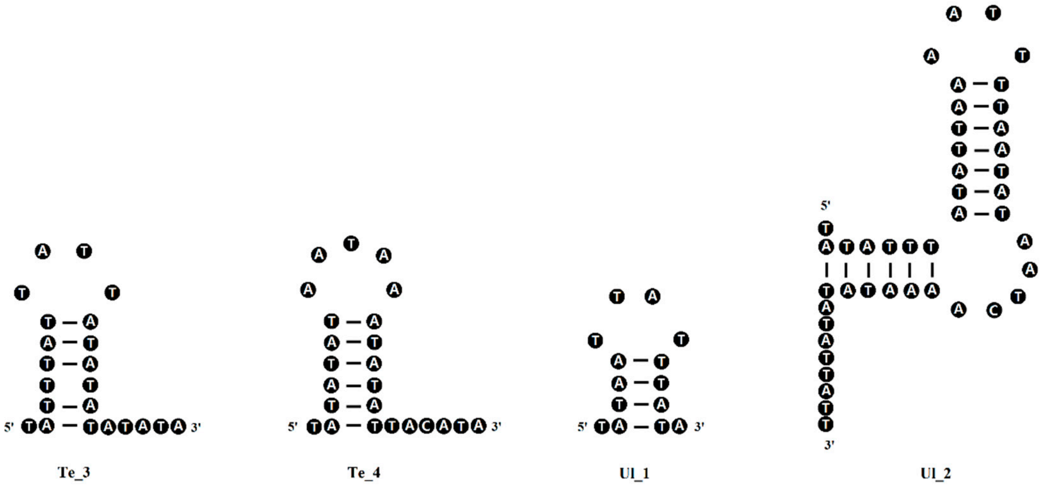

Figure S5. The putative secondary structure of four repeat unit sequences in the AT-rich region. Note: Bars denote Watson-Crick base pairings.

Table S1. The A + T content of different parts in ten Tenebrionidae mitogenomes.

Feature	*Ad*	*As*	*Te1*	*Te2*	*Ul*	*Tr1*	*Tr2*	*Tr3*	*Tr4*	*Tr5*
Whole mitogenome	72.7	69.7	72.4	72.2	72.1	71.7	71.7	71.8	72.8	73.6
Protein coding genes	70.4	66.9	69.3	69.1	69.6	69.2	69.2	69.3	70.9	71.6
The 1st codon sites	66.1	65.4	64.8	64.9	64.5	64.7	64.7	64.7	65.9	67.0
The 2nd codon sites	67.0	66.3	66.8	66.8	66.5	66.3	66.1	66.3	65.7	66.2
The 3rd codon sites	78.0	69.1	76.3	75.6	78.0	76.5	76.8	76.8	81.0	81.5
rRNA genes	78.0	74.5	78.3	78.4	77.3	76.1	76.2	76.1	77.4	76.9
tRNA genes	76.6	74.3	76.9	77.0	76.9	75.6	75.8	75.8	76.9	77.4
AT-rich region	79.3	80.5	85.4	85.1	83.7	82.5	82.3	82.9	79.5	81.4

Table S2. The AT-skew of different parts in ten Tenebrionidae mitogenomes.

Feature	*Ad*	*As*	*Te1*	*Te2*	*Ul*	*Tr1*	*Tr2*	*Tr3*	*Tr4*	*Tr5*
Whole mitogenome	0.14	0.20	0.20	0.20	0.14	0.11	0.11	0.11	0.13	0.11
PCG-J	0.03	0.10	0.10	0.09	0.00	−0.01	0.00	0.00	0.01	0.01
PCG1-J	0.13	0.21	0.20	0.20	0.15	0.14	0.14	0.14	0.16	0.14
PCG2-J	−0.36	−0.35	−0.37	−0.37	−0.38	−0.36	−0.36	−0.36	−0.37	−0.35
PCG3-J	0.26	0.44	0.41	0.40	0.21	0.18	0.20	0.19	0.20	0.19
PCG-N	−0.32	−0.38	−0.38	−0.38	−0.36	−0.29	−0.30	−0.30	−0.31	−0.28
PCG1-N	−0.12	−0.18	−0.12	−0.12	−0.19	−0.14	−0.14	−0.14	−0.18	−0.13
PCG2-N	−0.43	−0.44	−0.45	−0.45	−0.48	−0.47	−0.47	−0.47	−0.48	−0.46
PCG3-N	−0.39	−0.52	−0.54	−0.54	−0.41	−0.28	−0.28	−0.28	−0.28	−0.27
rRNA genes	−0.17	−0.19	−0.23	−0.22	−0.17	−0.13	−0.13	−0.13	−0.16	−0.13
tRNA-J	0.07	0.13	0.09	0.09	0.09	0.09	0.09	0.09	0.10	0.08
tRNA-N	−0.06	−0.09	−0.06	−0.06	−0.05	−0.06	−0.06	−0.06	−0.09	−0.06
AT-rich region	0.06	0.11	0.22	0.22	−0.02	0.02	0.02	0.03	0.00	0.02

AT skew = [A − T]/[A + T]; PCG1-3 denotes the three codon sites in PCGs; J and N denote the encoded strand, respectively.

Table S3. The GC-skew of different parts in ten Tenebrionidae mitogenomes.

Feature	*Ad*	*As*	*Te1*	*Te2*	*Ul*	*Tr1*	*Tr2*	*Tr3*	*Tr4*	*Tr5*
Whole mitogenome	−0.22	−0.36	−0.27	−0.27	−0.33	−0.31	−0.31	−0.31	−0.31	−0.27
PCG-J	−0.20	−0.36	−0.27	−0.26	−0.32	−0.29	−0.30	−0.29	−0.28	−0.25
PCG1-J	0.09	−0.03	0.04	0.05	−0.06	−0.02	−0.03	−0.02	−0.01	−0.01
PCG2-J	−0.27	−0.32	−0.29	−0.29	−0.28	−0.28	−0.28	−0.28	−0.27	−0.28
PCG3-J	−0.54	−0.78	−0.69	−0.67	−0.82	−0.69	−0.74	−0.73	−0.77	−0.62
PCG-N	0.24	0.41	0.28	0.28	0.37	0.35	0.36	0.36	0.36	0.32
PCG1-N	0.33	0.45	0.28	0.28	0.41	0.42	0.41	0.41	0.47	0.45
PCG2-N	0.03	0.04	0.04	0.04	0.04	0.08	0.08	0.08	0.08	0.08
PCG3-N	0.45	0.75	0.62	0.60	0.82	0.69	0.73	0.73	0.73	0.57
rRNA genes	0.31	0.39	0.34	0.34	0.40	0.40	0.39	0.39	0.39	0.38
tRNA-J	0.01	−0.05	0.00	0.01	−0.02	−0.04	−0.04	−0.04	−0.03	−0.03
tRNA-N	0.36	0.38	0.41	0.41	0.43	0.42	0.42	0.42	0.42	0.42
AT-rich region	−0.17	−0.36	−0.39	−0.34	−0.22	−0.20	−0.18	−0.19	−0.36	−0.19

GC skew = [G − C]/[G + C]; PCG1-3 denotes the three codon sites in PCGs; J and N denote the encoded strand, respectively.

Table S4. The A + T content of each protein coding gene in ten Tenebrionidae mitogenomes.

Feature	*Ad*	*As*	*Te1*	*Te2*	*Ul*	*Tr1*	*Tr2*	*Tr3*	*Tr4*	*Tr5*
ATP6	70.4	65.6	67.7	68.0	67.7	68.5	68.8	68.6	68.9	71.2
ATP8	75.0	68.6	76.5	76.5	75.2	81.7	81.4	82.1	74.2	73.7
COX1	63.1	61.6	62.0	61.4	64.1	61.3	61.1	61.4	64.8	64.3
COX2	68.4	63.5	66.1	66.7	67.4	66.4	65.9	66.2	68.1	69.7
COX3	66.9	62.8	64.1	64.2	65.3	66.3	66.5	66.8	68.5	68.2
CYTB	66.2	63.1	66.3	66.4	64.9	64.8	65.0	64.7	67.0	69.3
ND1	73.2	70.0	71.4	71.2	71.7	72.5	72.9	72.9	73.0	75.2
ND2	72.1	69.0	73.9	73.8	74.0	72.1	71.5	71.4	71.5	72.2
ND3	69.8	65.2	67.8	68.1	68.7	70.6	70.0	70.6	71.8	72.4
ND4	73.7	69.5	72.1	71.8	72.5	72.3	72.4	72.4	74.5	74.4
ND4L	76.8	70.9	74.7	73.3	72.0	74.4	74.4	74.0	77.2	77.5
ND5	73.9	71.2	72.8	72.5	73.5	72.9	73.0	73.1	74.5	74.8
ND6	75.4	71.8	76.0	75.2	74.6	72.2	72.6	72.8	75.6	76.0

Table S5. The AT-skew of each protein coding gene in ten Tenebrionidae mitogenomes.

Feature	*Ad*	*As*	*Te1*	*Te2*	*Ul*	*Tr1*	*Tr2*	*Tr3*	*Tr4*	*Tr5*
ATP6	−0.03	0.09	0.07	0.07	−0.04	−0.01	0.00	0.00	−0.02	0.00
ATP8	0.18	0.29	0.25	0.25	0.10	0.10	0.12	0.11	0.10	0.06
COX1	0.01	0.04	0.05	0.05	−0.02	−0.02	−0.02	−0.02	−0.01	0.01
COX2	0.08	0.11	0.11	0.10	0.05	0.12	0.12	0.12	0.06	0.09
COX3	−0.02	0.10	0.06	0.06	−0.03	−0.06	−0.04	−0.04	−0.03	−0.03
CYTB	−0.01	0.11	0.10	0.09	0.00	−0.02	−0.02	−0.02	−0.02	−0.02
ND1	−0.36	−0.41	−0.41	−0.41	−0.41	−0.33	−0.33	−0.33	−0.34	−0.32
ND2	0.09	0.13	0.13	0.13	0.06	0.01	0.03	0.03	0.06	0.03
ND3	−0.05	0.07	0.02	0.01	−0.08	−0.09	−0.08	−0.09	−0.04	−0.06
ND4	−0.35	−0.40	−0.42	−0.42	−0.41	−0.31	−0.31	−0.31	−0.37	−0.31
ND4L	−0.38	−0.42	−0.37	−0.40	−0.36	−0.30	−0.29	−0.29	−0.27	−0.31
ND5	−0.29	−0.37	−0.35	−0.36	−0.32	−0.28	−0.28	−0.28	−0.28	−0.26
ND6	0.08	0.20	0.20	0.20	0.05	−0.04	−0.04	−0.04	0.09	−0.03

AT skew = [A − T]/[A + T].

Table S6. The GC-skew of each protein coding gene in ten Tenebrionidae mitogenomes.

Feature	*Ad*	*As*	*Te1*	*Te2*	*Ul*	*Tr1*	*Tr2*	*Tr3*	*Tr4*	*Tr5*
ATP6	−0.21	−0.37	−0.29	−0.28	−0.37	−0.36	−0.37	−0.37	−0.35	−0.34
ATP8	−0.49	−0.76	−0.56	−0.56	−0.58	−0.43	−0.45	−0.43	−0.56	−0.71
COX1	−0.11	−0.21	−0.16	−0.16	−0.16	−0.19	−0.19	−0.19	−0.13	−0.13
COX2	−0.20	−0.31	−0.26	−0.26	−0.37	−0.33	−0.32	−0.32	−0.28	−0.24
COX3	−0.13	−0.31	−0.23	−0.22	−0.24	−0.13	−0.15	−0.15	−0.20	−0.18
CYTB	−0.22	−0.34	−0.25	−0.24	−0.32	−0.31	−0.32	−0.31	−0.24	−0.23
ND1	0.28	0.39	0.27	0.27	0.37	0.27	0.29	0.28	0.34	0.23
ND2	−0.27	−0.51	−0.41	−0.40	−0.42	−0.37	−0.41	−0.40	−0.44	−0.33
ND3	−0.32	−0.49	−0.33	−0.32	−0.44	−0.39	−0.40	−0.39	−0.45	−0.36
ND4	0.20	0.43	0.28	0.26	0.35	0.36	0.36	0.35	0.36	0.32
ND4L	0.36	0.52	0.31	0.32	0.54	0.40	0.37	0.35	0.57	0.38
ND5	0.24	0.39	0.27	0.29	0.36	0.37	0.38	0.38	0.35	0.34
ND6	−0.34	−0.62	−0.51	−0.48	−0.57	−0.47	−0.50	−0.49	−0.45	−0.46

GC skew = [G − C]/[G + C].

Table S7. Initiation codons in each protein coding gene of ten Tenebrionidae mitogenomes.

Species	*ATP6*	*ATP8*	*COX1*	*COX2*	*COX3*	*CYTB*	*ND2*	*ND3*	*ND6*	*ND1*	*ND4*	*ND4L*	*ND5*
Ad	ATG	ATA	CCG	ATA	ATG	ATG	ATA	TTG	ATC	ATA	ATA	ATG	ATT
As	ATG	ATT	CCG	ATA	ATG	ATG	ATA	ATC	ATA	ATA	ATG	ATG	ATT
Te1	ATG	ATA	CCG	ATA	ATG	ATG	ATC	ATT	ATT	ATA	ATG	ATG	ATT
Te2	ATG	ATA	CCG	ATA	ATG	ATG	ATC	ATT	ATT	ATA	ATG	ATG	ATT
Ul	ATG	ATT	CCG	ATA	ATG	ATG	ATT	ATT	ATC	ATA	ATG	ATG	TTG
Tr1	ATG	ATT	CTG	ATA	ATG	ATG	ATA	ATA	ATC	ATA	ATG	ATG	ATT
Tr2	ATG	ATT	CTG	ATA	ATG	ATG	ATA	ATA	ATC	ATA	ATG	ATG	ATT
Tr3	ATG	ATT	CTG	ATA	ATG	ATG	ATA	ATA	ATC	ATA	ATG	ATG	ATT
Tr4	ATG	ATA	CCG	ATA	ATG	ATG	ATT	ATA	ATA	ATA	ATG	ATG	ATT
Tr5	ATG	ATC	CTG	ATA	ATG	ATG	ATA	ATT	ATT	ATA	ATG	ATG	ATT

Table S8. Termination codons in each protein coding gene of ten Tenebrionidae mitogenomes.

Species	*ATP6*	*ATP8*	*COX1*	*COX2*	*COX3*	*CYTB*	*ND2*	*ND3*	*ND6*	*ND1*	*ND4*	*ND4L*	*ND5*
Ad	TAA	TAA	T	T	T	TAA	TAA	TAG	TAA	TAG	TAA	TAA	T
As	TAA	TAA	T	T	T	TAA	TAA	TAG	TAA	TAG	TAA	TAA	T
Te1	TAA	TAA	TAA	T	T	TAA	TAA	TAG	TAA	TAG	TA	TAA	T
Te2	TAA	TAA	TAA	T	T	TAA	TAA	TAG	TAA	TAG	TA	TAA	T
Ul	TAA	TAG	T	T	TAA	TAA	TAA	TAG	TAA	TAG	TA	TAA	T
Tr1	TAA	TAG	TAA	TAA	TAA	TAA	TAA	TAG	TAA	TAG	TA	TAA	T
Tr2	TAA	TAG	TAA	T	TAA	TAA	TAA	TAG	TAA	TAG	TA	TAA	T
Tr3	TAA	TAG	TAA	T	TAA	TAA	TAA	TAG	TAA	TAG	TA	TAA	T
Tr4	TAA	TAG	T	T	T	TAA	TAG	TAA	TAA	TAG	TA	TAA	T
Tr5	TAA	TAG	TAA	T	TAA	TAA	TAA	TAA	TAA	TAG	TA	TAA	T

Table S9. The usage rate of four most frequently used codons in ten Tenebrionidae mitogenomes.

Species	UUU(F)	UUA(L)	AUU(I)	AUA(M)	SUM	Total	Rate
Ad	298	310	307	243	1158	4264	27.2%
As	247	221	248	211	927	4267	21.7%
Te1	289	225	328	275	1117	4273	26.1%
Te2	293	229	325	266	1113	4273	26.0%
Ul	299	293	331	189	1112	4263	26.1%
Tr1	295	291	366	204	1156	4265	27.1%
Tr2	293	292	368	204	1157	4267	27.1%
Tr3	297	287	365	205	1154	4265	27.1%
Tr4	295	355	347	234	1231	4267	28.8%
Tr5	289	357	369	230	1245	4268	29.2%

Table S10. The usage rate of codons ended with different nucleotides.

Feature	NNU	NNC	NNA	NNG
PCG	38.0%	14.2%	39.0%	8.8%
PCG-J	27.6%	21.1%	47.7%	3.6%
PCG-N	54.5%	3.2%	25.0%	17.2%

Table S11. The size of 22 tRNA genes and two rRNA genes in ten Tenebrionidae mitogenomes.

Feature	*Ad*	*As*	*Te1*	*Te2*	*Ul*	*Tr1*	*Tr2*	*Tr3*	*Tr4*	*Tr5*	AVE	STDEV	%INUC
trnI	64	64	64	64	64	63	63	63	63	63	64	0.5	77.3
trnQ	69	69	69	69	69	69	69	69	69	69	69	0.0	53.6
trnM	66	69	68	68	68	68	68	68	68	68	68	0.7	79.7
trnW	64	68	66	66	64	67	67	67	64	67	66	1.5	75.4
trnC	62	61	61	61	65	61	61	61	62	61	62	1.3	57.6
trnY	64	64	66	66	63	65	64	64	65	66	65	1.1	47.1
trnL^{UUR}	65	64	65	65	65	65	65	65	66	68	65	1.1	73.5
trnK	70	71	70	70	70	71	71	71	71	71	71	0.5	73.6
trnD	64	65	65	65	66	66	66	66	66	68	66	1.1	50.0
trnG	62	63	62	62	63	62	62	62	64	65	63	1.1	63.6
trnA	65	67	66	66	66	67	67	67	65	67	66	0.8	69.1
trnR	63	66	64	64	64	63	63	63	68	65	64	1.6	54.4
trnN	66	66	65	65	65	64	64	64	66	65	65	0.8	76.1
trnS^{AGN}	58	60	60	60	60	59	59	59	60	59	59	0.7	76.2
trnE	62	64	62	62	62	64	65	65	63	64	63	1.3	66.2
trnF	64	64	64	64	68	65	65	65	65	67	65	1.4	50.0
trnH	63	64	63	63	65	65	65	65	65	65	64	0.9	51.5
trnT	63	62	63	63	63	63	63	63	63	63	63	0.3	69.8
trnP	64	63	66	66	64	66	66	66	66	67	65	1.3	67.6
trnS^{UCN}	67	67	66	66	68	68	68	68	67	67	67	0.8	66.7
trnL^{CUN}	62	62	65	65	65	64	64	64	66	65	64	1.3	71.2
trnV	69	69	69	69	68	69	69	69	68	69	69	0.4	78.3
16S rRNA	1280	1279	1280	1281	1279	1280	1281	1282	1277	1288	1281	2.9	54.3
12S rRNA	762	747	761	761	764	754	754	754	759	760	758	5.2	57.6

AVE denotes the average size of each RNA; STDEV denotes standard deviation of the RNA size from different Tenebrionidae mitogenomes; %INUC denotes the identical percent of nucleotide in each RNA.

Table S12. The identical percent of nucleotide in each part of the secondary structure of tRNAs in ten Tenebrionidae mitogenomes.

Feature	AA Stem	DHU Arm	AC Arm	TψC Arm	D-loop	AC Loop	Variable Loop	TψC Loop
trnI	93.33%	100.00%	90.00%	75.00%	80.00%	100%	40.00%	0.00%
trnQ	46.67%	87.50%	0.00%	40.00%	80.00%	100%	50.00%	85.71%
trnM	86.67%	100.00%	70.00%	100.00%	66.67%	100%	50.00%	50.00%
trnW	80.00%	100.00%	90.00%	75.00%	37.50%	100%	75.00%	0.00%
trnC	66.67%	75.00%	60.00%	50.00%	25.00%	85.71%	75.00%	25.00%
trnY	40.00%	66.67%	70.00%	62.50%	37.50%	100%	25.00%	0.00%
trnL^{UUR}	93.33%	100.00%	60.00%	100.00%	83.33%	71.43%	50.00%	16.67%
trnK	86.67%	66.67%	100%	60.00%	100%	100%	33.33%	57.14%
trnD	53.33%	87.50%	30.00%	50.00%	40.00%	71.43%	50.00%	20.00%
trnG	33.33%	100.00%	60.00%	100.00%	71.43%	100%	100.00%	50.00%
trnA	93.33%	100.00%	100%	37.50%	50.00%	71.43%	75.00%	0.00%
trnR	53.33%	66.67%	60.00%	66.67%	14.29%	100%	60.00%	0.00%
trnN	93.33%	100.00%	70.00%	100.00%	75.00%	100%	40.00%	0.00%
trnS^{AGN}	100.00%		90.00%	100.00%		100%	25.00%	50.00%
trnE	60.00%	100.00%	80.00%	33.33%	40.00%	85.71%	75.00%	0.00%
trnF	60.00%	87.50%	30.00%	33.33%	33.33%	100%	50.00%	0.00%
trnH	53.33%	75.00%	20.00%	50.00%	60.00%	85.71%	75.00%	0.00%
trnT	73.33%	100.00%	70.00%	33.33%	60.00%	100%	75.00%	0.00%
trnP	73.33%	100.00%	80.00%	25.00%	80.00%	100%	50.00%	0.00%
trnS^{UCN}	93.33%	50.00%	20.00%	75.00%	60.00%	85.71%	50.00%	33.33%
trnL^{CUN}	73.33%	100.00%	50.00%	62.50%	83.33%	100%	75.00%	16.67%
trnV	93.33%	75.00%	100%	70.00%	40.00%	85.71%	25.00%	71.43%
AVE	72.73%	87.50%	63.64%	63.60%	57.97%	92.86%	55.61%	21.63%

Table S13. The identical percent of nucleotide in each helix of the secondary structure of *12S rRNA* in ten Tenebrionidae mitogenomes.

Domain	Helix	Total Site	Conserved Site	Percentage of Conserved Site
I	H9	10	9	90.00%
	H17	6	1	16.67%
	H27	18	6	33.33%
	H39	10	4	40.00%
	H47	59	14	23.73%
	H367	31	16	51.61%
	H500	10	1	10.00%
	H511	26	21	80.77%
II	H567	6	1	16.67%
	H577	32	14	43.75%
	H673	39	8	20.51%
	H769	33	20	60.61%
	H885	22	14	63.64%

Table S13. *Cont.*

Domain	Helix	Total Site	Conserved Site	Percentage of Conserved Site
III	H921	35	34	97.14%
	H939	10	10	100.00%
	H944	25	21	84.00%
	H960	8	7	87.50%
	H984	14	5	35.71%
	H1047	40	31	77.50%
	H1068	12	8	66.67%
	H1074	6	4	66.67%
	H1113	8	0	0.00%
	H1241	4	1	25.00%
	H1303	16	6	37.50%
	H1350	18	6	33.33%
IV	H1399	34	27	79.41%
	H1506	20	16	80.00%

Table S14. The identical percent of nucleotide in each helix of the secondary structure of *16S rRNA* in ten Tenebrionidae mitogenomes.

Domain	Helix	Total Site	Conserved Site	Percentage of Conserved Site
I	H183	6	2	33.33%
	H235	18	12	66.67%
	H461	4	0	0.00%
	H533	6	0	0.00%
	H563	6	6	100.00%
II	H579	12	8	66.67%
	H589	12	5	41.67%
	H671	29	18	62.07%
	H687	31	6	19.35%
	H736	8	6	75.00%
	H777	6	6	100.00%
	H812	12	3	25.00%
	H822	10	6	60.00%
	H837	20	2	10.00%
	H946	20	6	30.00%
	H991	21	4	19.05%
	H1196	21	3	14.29%
	H1507	12	10	83.33%
	H1807	10	3	30.00%
IV	H1648	39	15	38.46%
	H1764	19	9	47.37%
	H1775	6	6	100.00%
	H1792	10	7	70.00%
	H1830	8	6	75.00%
	H1835	20	14	70.00%
	H1906	12	12	100.00%
	H1925	2	2	100.00%
	H1935	12	8	66.67%

Table S14. *Cont.*

Domain	Helix	Total Site	Conserved Site	Percentage of Conserved Site
V	H2023	10	8	80.00%
	H2043	24	18	75.00%
	H2064	25	24	96.00%
	H2077	50	6	12.00%
	H2246	10	5	50.00%
	H2259	12	6	50.00%
	H2347	16	7	43.75%
	H2395	8	8	100.00%
	H2455	35	28	80.00%
	H2507	27	27	100.00%
	H2520	19	14	73.68%
	H2547	10	7	70.00%
	H2588	15	12	80.00%
VI	H2646	16	6	37.50%
	H2675	8	4	50.00%
	H2735	4	4	100.00%

Table S15. Intergenic gaps and overlaps in ten Tenebrionidae mitogenomes.

Feature	Gene Gaps and Overlaps									
	Ad	***As***	***Te1***	***Te2***	***Ul***	***Tr1***	***Tr2***	***Tr3***	***Tr4***	***Tr5***
Genome size	16449	15828	15785	15784	15434	15881	15883	15876	15813	15925
trnI-trnQ	−3	−3	−3	−3	6	−3	−3	−3	−3	−3
trnQ-trnM	−1	−1	−1	−1	0	−1	−1	−1	−1	−1
trnM-ND2	0	0	−15	−15	6	6	6	6	6	6
ND2-trnW	−2	−2	−2	−2	6	−2	−2	−2	1	−2
trnW-trnC	−1	−1	22	22	12	−1	−1	−1	−1	−1
trnC-trnY	0	0	0	0	10	2	2	2	0	1
trnY-COX1	1	1	1	1	1	7	7	7	1	2
COX1-trnL(UUR)	0	0	−5	−5	0	1	1	1	0	1
trnL(UUR)-COX2	0	0	0	0	0	0	0	0	0	0
COX2-trnK	0	0	0	0	0	1	0	0	0	0
trnK-trnD	−1	−1	−1	−1	−1	33	33	31	48	12
trnD-ATP8	732	0	0	0	0	0	0	0	0	0
ATP8-ATP6	−7	−7	−7	−7	−7	−7	−7	−7	−7	−7
ATP6-COX3	−1	−1	−1	−1	−1	−1	−1	−1	−1	−1
COX 3-trnG	0	0	0	0	0	2	2	2	0	0
trnG-ND3	0	0	0	0	0	−7	−7	−7	0	0
ND3-trnA	−2	−2	−2	−2	−2	−2	−2	−2	2	2
trnA-trnR	−1	−1	−1	−1	−1	−1	−1	−1	−1	−1
trnR-trnN	−1	−1	−1	−1	−1	−1	−1	−1	−1	−1
trnN-trnS(AGN)	0	0	0	0	0	0	0	0	0	0
trnS(AGN)-trnE	0	0	1	1	0	0	0	0	0	0
trnE-trnF	−2	−2	−2	−2	−2	−2	−2	−2	−2	−2
trnF-ND5	0	0	0	0	0	0	0	0	0	0
ND5-trnH	0	0	0	0	1	0	0	0	0	0
trnH-ND4	−1	−1	−1	−1	−1	−1	−1	−1	−1	−1
ND4-ND4L	−4	−7	−7	−7	−7	−7	−7	−7	−7	−7
ND4L-trnT	2	2	2	2	2	2	2	2	2	2
trnT-trnP	0	0	0	0	0	0	0	0	0	0
trnP-ND6	2	2	2	2	2	2	2	2	2	2
ND6-CYTB	−1	−1	−1	−1	−1	−1	−1	−1	−1	−1
CYTB-trnS(UCN)	−2	−2	−2	−2	−2	−2	−2	−2	−2	−2
trnS(UCN)-ND1	17	17	17	17	18	17	17	17	17	17
ND1-trnL(CUN)	−3	−3	−3	−3	−3	−3	−3	−3	−6	−3
trnV-12SrRNA	11	2	12	21	4	21	21	2	15	21
AT-rich region	1157	1244	1170	1160	798	1239	1237	1256	1147	1266

Numbers with "−" denote gene overlaps; those without "−" denote gene gaps.
